# The Crosstalk Between Regulatory Non-Coding RNAs and Nuclear Factor Kappa B in Hepatocellular Carcinoma

**DOI:** 10.3389/fonc.2021.775250

**Published:** 2021-11-05

**Authors:** Yina Zhang, Jiajia Shao, Shuangshuang Li, Yanning Liu, Min Zheng

**Affiliations:** State Key Laboratory for Diagnosis and Treatment of Infectious Diseases, National Clinical Research Center for Infectious Diseases, Collaborative Innovation Center for Diagnosis and Treatment of Infectious Diseases, The First Affiliated Hospital, College of Medicine, Zhejiang University, Hangzhou, China

**Keywords:** nuclear factor kappa B, hepatocellular carcinoma, microRNA, long non-coding RNA, chemoresistance

## Abstract

Hepatocellular carcinoma (HCC) is a highly lethal type of malignancies that possesses great loss of life safety to human beings worldwide. However, few effective means of curing HCC exist and its specific molecular basis is still far from being fully elucidated. Activation of nuclear factor kappa B (NF-κB), which is often observed in HCC, is considered to play a significant part in hepatocarcinogenesis and development. The emergence of regulatory non-coding RNAs (ncRNAs), particularly microRNAs (miRNAs) and long non-coding RNAs (lncRNAs), is a defining advance in cancer biology, and related research in this branch has yielded many diagnostic and therapeutic opportunities. Recent studies have suggested that regulatory ncRNAs act as inhibitors or activators in the initiation and progression of HCC by targeting components of NF-κB signaling or regulating NF-κB activity. In this review, we attach importance to the role and function of regulatory ncRNAs in NF-κB signaling of HCC and NF-κB-associated chemoresistance in HCC, then propose future research directions and challenges of regulatory ncRNAs mediated-regulation of NF-κB pathway in HCC.

## Introduction

As the most important intracellular nuclear transcription factor, the nuclear factor kappa B (NF-κB) promotes the transcription of genes with κB binding sites that are responsible for the manipulation of multiple biological processes, such as inflammation, immune response, and apoptosis (1). Recently, it has been reported that constitutive activation of the NF-κB signaling is observed in hepatocellular carcinoma (HCC) (2, 3). Additionally, accumulating evidence has shown that NF-κB plays a critical role in the transcriptional regulation of genes that concern diverse pathological aspects of HCC with respect to cell transformation, proliferation, survival, invasion, metastasis and drug resistance (4–6). HCC that accounts for 80%-90% of all primary liver cancers is ranked as the second leading cause of cancer-related deaths and the fifth most common human cancer around the world (7–10). Therefore, targeting NF-κB signaling pathway warrants future research, which may contribute to novel HCC-specific diagnostic and therapeutic strategies.

NF-κB is assembled into a heterodimeric or homodimeric complex by different subunits of the Rel family, which consists of five members, including RelA (p65), RelB, C-Rel, NF-κB1 (p50/p105), and NF-κB2 (p52/p100) (11). Under physiological conditions, these subunits are associated with the inhibitor of κB (IκB), whose function is to effectively sequester NF-κB in the cytoplasm. When cells are stimulated by a cascade of signaling events such as stress, bacteria, viruses or cytokines, NF-κB becomes rapidly activated, then translocates into the nucleus where it binds to the κB elements of gene promoters or enhancers, thereby triggering transcription of target genes (12). Typically, there are two different pathways that mediate NF-κB activation, including a canonical and a noncanonical pathway. In the canonical pathway, the key event is the release of NF-κB from the NF-κB/IκB trimer. In response to specific stimuli, NF-κB-bound IκB is phosphorylated at Ser32 and Ser36 residues *via* the IκB kinase (IKK) complex formed by two catalytic subunits (IKK1/2, a.k.a. IKKα and IKKβ) and the scaffold/adaptor protein NF-κB essential modulator (NEMO; also known as IKKγ) (13). Phosphorylated IκB subsequently quickly undergoes polyubiquitination through the SCF-β-TrCP complex followed by 26S proteasome-mediated degradation, allowing the nucleus entry of NF-κB (14–16). The noncanonical NF-κB pathway, which is usually activated following the induction of members of the tumour necrosis factor receptor (TNFR) superfamily, mainly relies on NF-κB-induced kinase (NIK) and IKKα subunits to induce phosphorylation of NF-κB precursor protein p100 at Ser866 and Ser870 residues (14, 17). Phosphorylation targets p100 for subsequent partial processing to form the mature NF-κB p52 subunit through the ubiquitin-proteasome pathway, which then binds to RelB to form a p52-RelB heterodimer with transcriptional activity (17).

**Figure 1:**
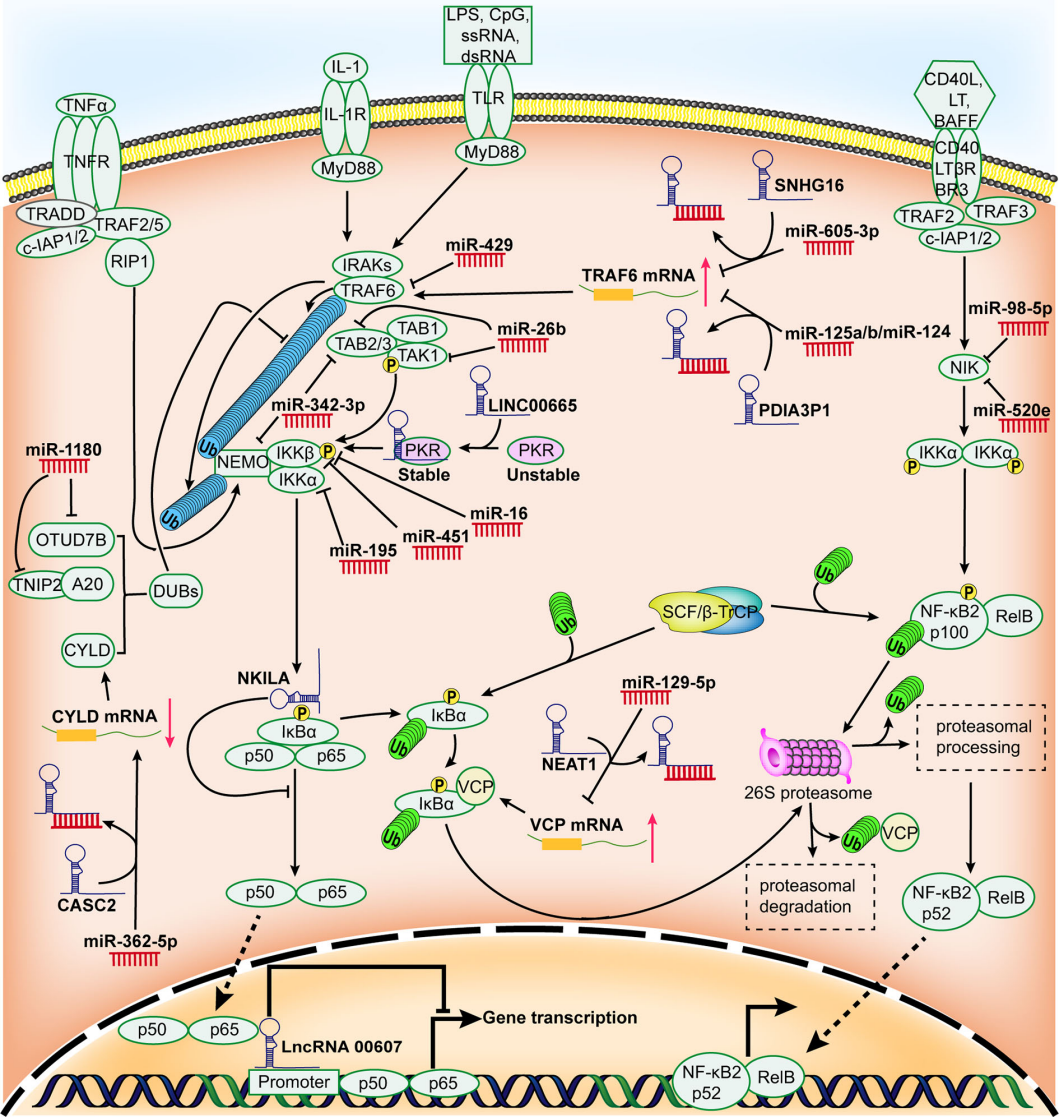
Schematic representation for the role of regulatory ncRNAs in NF-κB signaling pathway in HCC. Many miRNAs and lncRNAs are aberrantly expressed in HCC and they can promote or restrain the expression of HCC-associated genes by modulating certain components of NF-κB signaling pathway and/or the activity of NF-κB.

Apart from the common pathways described above, recent evidence indicates that non-coding RNAs (ncRNAs) act as vital regulatory roles in the NF-κB signaling by diverse mechanisms. Despite a lack of protein-coding potential, ncRNAs serve as pivotal functional components or regulatory molecules for genetic expression (18). Generally, ncRNAs can be categorized into housekeeping ncRNAs and regulatory ncRNAs in term of their discrepancy in expression levels and functional features. The former that are profusely and omnipresently expressed in cells are necessary for cells to survive, the latter usually participate in genetic expression at epigenetic, transcriptional, and post-transcriptional levels (19). Among regulatory ncRNAs, the regulation of NF-κB signaling in HCC by microRNAs (miRNAs) and long non-coding RNAs (lncRNAs) has been relatively well characterized, while other regulatory ncRNAs, for instance, small interfering RNAs (siRNAs), PIWI interacting RNAs (piRNAs) as well as circular RNAs (circRNAs), have been rarely reported to regulate NF-κB signaling of HCC. Herein, we put a particularly focus on up-to-date findings regarding the role of ncRNAs in NF-κB signaling of HCC (**Figure 1**), then discuss the potential significance of ncRNAs in overcoming the obstacle of NF-κB-associated chemoresistance in HCC, finally future research directions and challenges are addressed.

## Regulation of NF-κB Signaling by miRNAs in HCC

miRNAs are a distinct kind of evolutionarily-conserved and endogenous ncRNAs of 19–25 nt in length (20). The predominant function of miRNAs includes either accelerated degradation or reduced translation of target messenger RNAs (mRNAs), which can be achieved by the conjugation of a miRNA to the 3 ‘untranslated region (UTR) of the target mRNA (21–23). miRNAs have recently acquired considerable attention in the research of hepatic carcinoma (24). Overwhelming evidence has emerged that miRNAs are involved in the malignant biological activity of HCC by playing the part of either oncogenic or tumor suppressor factors (25), some of which have been reported directly or indirectly to regulate NF-κB pathway and/or NF-κB activity to mediate HCC development (**Table 1** and **Figure 1**).

**Table 1:** miRNAs involved in the regulation of NF-κB pathway and/or NF-κB activity in HCC.

miRNA	Expression	Target	Function in NF-κB	Role in HCC	Ref.
miR-429	Down	TRAF6	NF-κB inhibition	HCC suppressor	(26)
miR-98-5p	Down	NIK	NF-κB inhibition	HCC suppressor	(27)
miR-520e	Down	NIK	NF-κB inhibition	HCC suppressor	(28)
miR-26b	Down	TAK1 and TAB3	NF-κB inhibition	HCC suppressor	(29)
miR-16	Down	IKKβ	NF-κB inhibition	HCC suppressor	(30)
miR-451	Down	IKKβ	NF-κB inhibition	HCC suppressor	(31)
miR-195	Down	IKKα	NF-κB inhibition	HCC suppressor	(32)
miR-342-3p	Down	IKKγ, TAB2 and TAB3	NF-κB inhibition	HCC suppressor	(33)
miR-127-5p	Down	BLVRB	NF-κB inhibition	HCC suppressor	(34)
miR-16	Down	FEAT	NF-κB inhibition	HCC suppressor	(35)
miR-194	Down	TRIM23	NF-κB inhibition	HCC suppressor	(36)
miR-370	Down	Lin28A	NF-κB inhibition	HCC suppressor	(37)
miR-129-5p	Down	VCP	NF-κB inhibition	HCC suppressor	(38)
miR-302b	Down	AKT2	NF-κB inhibition	HCC suppressor	(39)
miR-491	Down	SMAD3	NF-κB inhibition	HCC suppressor	(40)
miR-622	Down	MAP4K4	NF-κB inhibition	HCC suppressor	(41)
miR-29-3p	Down	PTEN	NF-κB inhibition	HCC suppressor	(42)
miR-595	Down	ABCB1	NF-κB inhibition	HCC suppressor	(43)
miR-140	Down	Dnmt1	NF-κB inhibition	HCC suppressor	(44)
miR-124	Down	BIRC3	NF-κB inhibition	HCC suppressor	(45)
miR-145	Down	ROCK1	NF-κB inhibition	HCC suppressor	(46)
miR-301a	Up	GAX	NF-κB activation	Oncogene	(47)
miR-4262	Up	PDCD4	NF-κB activation	Oncogene	(48)
miR-657	Up	TLE1	NF-κB activation	Oncogene	(49)
miR-1180	Up	OTUD7B and TNIP2	NF-κB activation	Oncogene	(50)
miR-362-5p	Up	CYLD	NF-κB activation	Oncogene	(51)

TRAF6, Tumor necrosis factor receptor-associated factor 6; TAK1, Transforming growth factor-β-activated kinase 1; TAB3, TAK-binding protein 3; BLVRB, Biliverdin reductase B; TRIM23, Transcripts encoding tripartite motif containing 23; Lin28A, Lin28 homolog A; VCP, Valosin containing protein; MAP4K4, Mitogen-activated protein 4 kinase 4; Dnmt1, DNA methyltransferase 1; BIRC3, Baculoviral IAP Repeat Containing 3; ROCK1, Rho-associated protein kinase 1; TLE1, Transducin-like Enhancer Protein 1.

### miRNAs Involved in the Regulation of TRAFs

TRAFs are important signaling molecules that connect the TNFR superfamily and the interleukin-1 receptor/Toll-like receptor (IL-1R/TLR) superfamily, which act as an active part in regulating immunity and inflammation (52). Recently, several evidence has demonstrated that TRAF proteins initiating NF-κB activation is regulated by ncRNAs in HCC and these studies focus mainly on TRAF6. TRAF6 is a well-characterized E3 ligase that specifically conjugates K63-linked polyubiquitin chains (53) and it is also considered as a key activator of NF-κB signaling (54). It was reported that the expression of TRAF6 was strongly associated with HCC oncogenicity both *in vitro* and *in vivo*. However, miR-429 (26), miR-125a/b/miR-124 (55) and miR-605-3p (56) were confirmed to dampen the expression of NF-κB target genes by targeting TRAF6, which significantly abrogated the malignancy of HCC. In addition, several studies have reported that other TRAF proteins, such as TRAF2, can be regulated by certain miRNA molecules such as miR-502-5p (57), miR-514a-3p (58), and miR-892b (59) in breast cancer, suggesting that these miRNAs that regulate TRAF2 can serve as potential disquisitive objects in the study of HCC development and progression.

### miRNAs Involved in the Regulation of NIK

NIK is a member of the mitogen-activated protein kinase kinase kinaser (MAPKKK, MAP3K) family, which is a central signaling component in the noncanonical NF-κB pathway (60, 61). Previous studies have indicated that the activation of the noncanonical NF-κB pathway by NIK significantly enhances oncogenic signaling and high NIK activity is associated with different human malignancies and supports poor survival in tumor patients (62). It is worth noting that NIK is identified as an underlying and attractive candidate for the treatment of HCC. Recently, silencing NIK with miRNAs has been acknowledged as an effective strategy for attenuating the constitutive activation of NF-κB in HCC. For example, miR-98-5p was confirmed to be a potent inhibitor of NF-κB pathway *via* markedly repressing NIK and exerted its inhibitory effect for anti-HCC therapy (27). Another study found that over-expression of miR-520e stunted HCC cells growth *via* reducing NIK protein levels (28).

### miRNAs Involved in the Regulation of TAK1

TAK1 is a serine/threonine protein kinase and is also an identified MAP3K. It is an important adaptor protein for intracellular signaling transduction that responds to TGF-β, bone morphogenetic proteins, and other cytokines (63, 64). These cytokines initially act on the corresponding cell surface receptors and then lead to the recruitment of the TRAF proteins in the cytoplasm to the receptors. TAK1 functions as a pivotal downstream kinase that mediates TRAF6-induced NF-κB pathway by forming a complex with the TAK-binding proteins (TAB 1, 2, and 3) (65). The complex then phosphorylates IKK complex to activate NF-κB pathway (54, 66). Recently, the potential linkage between miRNAs and TAK1 has been investigated. It was reported that the levels of miRNA-26b were dramatically decreased in HCC tissues, and enhancing miR-26b expression possessed the NF-κB inhibitory effect *via* targeting TAK1 and TAB3, thus attenuating HCC progression (29).

### miRNAs Involved in the Regulation of IKK

Recently, a plethora of studies have uncovered that the dysregulation of miRNAs may influence IKK, a key component of the canonical NF-κB pathway, thus triggering HCC initiation and progression. For example, the negative regulatory role for miR-16 has recently been discovered in HCC, and IKKβ is further characterized as a functional target of miR-16 (30). miR-451 is a key factor involved in the normal function of the liver and the loss of miR-451 is closely related to HCC progression (67, 68). Furthermore, a study reported that miR-451 strongly alleviated HCC cell proliferation through the direct suppression of IKKβ, thus downregulating the downstream genes of NF-κB pathway (31). miR-195 is a major member of the miR-15/16/195/424/497 family. At the molecular level, it is reported that miR-195 is able to modulate a large number of target proteins involved in cell cycle, apoptosis and proliferation (69). In addition, miR-195 is implicated in HCC pathogenesis by targeting IKK. miR-195 was shown to be markedly downregulated in HCC, and restoring the expression of miR-195 enabled it to regain its tumor suppressive function by affecting NF-κB downstream effectors by way of directly targeting IKKα and TAB3 at the post-transcriptional level (32). Another example was that increasing the expression of miR-342-3p was conducive to an evident decrease of proliferation level of HCC cells by directly targeting IKKγ, TAB2 and TAB3 3’UTR (33).

### miRNAs Involved in the Regulation of Deubiquitinating Enzymes (DUBs)

CYLD、A20、OTUD7B are well-known DUBs that play pivotal roles as negative regulators of the NF-κB pathway by blocking ubiquitination mediated by E3 ubiquitin ligases (70, 71). In addition, a previous study revealed that TNIP2 (also known as ABIN2), the binding partner of zinc finger protein A20, could impair NF-κB activation (72). miR-1180 was found to exert an anti-apoptotic function in HCC *via* directly targeting two NF-κB-negative regulators (OTUD7B and TNIP2), favouring NF-κB signaling activation (50). miR-362-5p was also confirmed to promote sustained NF-κB signaling activation through the suppression of CYLD, so as to aggravate HCC growth and metastasis (51, 73).

### miRNAs Involved in the Regulation of Other NF-κB-Associated Components

In HCC cells, the markedly under-expressed miR-127-5p led to increased activity of NF-κB by targeting BLVRB, thereby promoting the tumorigenicity (34). VCP was reported to be involved in the proteasome-mediated degradation of IκBα by physically interacting with ubiquitinated IκBα (74, 75). The overexpression of miR-129-5p was shown to negatively regulate the progression of HCC and inhibit the degradation of IκBα by suppressing the expression of VCP (38). On the contrary, some miRNAs are overexpressed in HCC and activate NF-κB activity by affecting certain NF-κB-associated factors, thereby predisposing to HCC development. For example, miR-4262 resulted in the accumulation of nuclear NF-κB/P65 by targeting the 3’UTR of PDCD4, which subsequently enhanced HCC cell proliferation (48). Similarly, miR-657 was proved to target TLE1 3’UTR, which in turn activated NF-κB signaling and conduced to HCC tumorigenesis (49). In short, the miRNA-NF-κB pathway network is expected to become a promising therapeutic target for patient with HCC.

## Regulation of NF-κB Signaling by lncRNAs in HCC

By convention, ncRNAs with the minimum size limit of 200 nt are defined as lncRNAs (76). Classification of lncRNAs is still at its infancy due to few structural, functional or mechanistic features common to all mammalian lncRNAs. Here, lncRNAs are categorized according to their modes of action and functions. Generally, the potential modes of action of lncRNAs depend on their subcellular localization. Functions of lncRNAs within the nucleus of the cell include transcriptional regulation, enhancer-associated ncRNAs, epigenetic regulation and regulation of nuclear architecture; Cytoplasmic functions of lncRNAs include targeting mRNAs for degradation by a process called Staufen 1 (STAU1)-mediated decay, maintaining mRNA stability and functioning as miRNA sponges (18). To date, lncRNAs have been revealed as essential regulators in HCC (77). Interestingly, some lncRNAs have been identified to implicate in hepatocarcinogenesis by modulating the NF-κB signaling (**Table 2** and **Figure 1**).

**Table 2:** LncRNAs involved in the regulation of NF-κB pathway and/or NF-κB activity in HCC.

LncRNA	Expression	Binding partners	Action modes	Function in NF-κB	Role in HCC	Ref.
miR503HG	Down	HNRNPA2B1	Protein ubiquitination and degradation	NF-κB inhibition	HCC suppressor	(75)
NKILA	Down	NF-κB/IκB complex	Protein stabilization	NF-κB inhibition	HCC suppressor	(76, 77)
CASC2	Down	miR-362-5p	miRNA sponge	NF-κB inhibition	HCC suppressor	(70)
00607	Down	the p65 promoter region	Transcriptional suppression	NF-κB inhibition	HCC suppressor	(78)
PDIA3P1	Up	miR-125a/b/miR-124	miRNA sponge	NF-κB activation	Oncogene	(55)
CRNDE	Up	miR-539-5p	miRNA sponge	NF-κB activation	Oncogene	(79)
SNHG16	Up	miR-17-5p	miRNA sponge	NF-κB activation	Oncogene	(80)
	Up	miR-605-3p	miRNA sponge	NF-κB activation	Oncogene	(56)
TP73-AS1	Up	miR-200a	miRNA sponge	NF-κB activation	Oncogene	(81)
Myd88	Up	N/A	Histone modification	NF-κB activation	Oncogene	(82)
SNHG12	Up	miR-199a/b-5p	miRNA sponge	NF-κB activation	Oncogene	(83)
LINC00665	Up	PKR	Protein activation and stabilization	NF-κB activation	Oncogene	(84)
NEAT1	Up	miR-129-5p	miRNA sponge	NF-κB activation	Oncogene	(85)

HNRNPA2B1, Heterogeneous nuclear ribonucleoprotein A2/B1; NKILA, NF-κB interacting lncRNA; CASC2, Cancer susceptibility candidate 2; PDIA3P1, Protein disulfide isomerase family A member 3 pseudogene 1; CRNDE, Colorectal Neoplasia Differentially Expressed; SNHG16, Small nucleolar RNA host gene 16; TP73-AS1, P73 antisense RNA 1 T; N/A, Not available; SNHG12, Small nucleolar RNA host gene 12; PKR, Double-stranded RNA (dsRNA)-activated protein kinase; NEAT1, Nuclear-enriched abundant transcript 1.

### LncRNAs Involved in the Regulation of NF-κB Pathway Through Transcription Regulation

Several studies have revealed that the lncRNAs-mediated regulation of NF-κB signaling in HCC can be in part attributed to lncRNA-DNA interplay. The typical lncRNA-DNA interaction site may be located in the promoters or other regulatory DNA sequences (such as enhancers) of certain genes, thus manipulating transcription of genes. For instance, lncRNA 00607 was able to bring about p65 transcriptional repression due to the interplay between lncRNA 00607 and NF-κB p65 promoter region, thus possessing attenuated proliferation of HCC cells (81). Furthermore, a portion of lncRNAs have been shown to participate in histone modification, revealing another pivotal transcriptional regulation mechanism. In terms of the regulation of NF-κB pathway in HCC, a study has demonstrated that the upregulation of lnc Myd88 in HCC contributes to the enrichment of acetylation of H3K27 at the promoter of Myd88, which promotes the transcription of Myd88 and then activates the NF-κB signaling pathway (85).

### LncRNAs Involved in the Regulation of NF-κB Pathway by Sponging miRNAs

Some lncRNAs act as competitive endogenous RNAs (ceRNAs) for miRNAs binding, and these lncRNAs are also hailed as miRNA sponges. This lncRNA-miRNA association reduces the levels of free miRNAs and weakens the “silencing effect” of miRNAs on target genes, thereby permitting the re-expression of the target genes of miRNAs (86). To date, a number of lncRNAs have been revealed as miRNA sponges involving in diverse pathological aspects of HCC by regulating NF-κB signaling pathway, among which tumor-suppressor lncRNAs negatively regulate the NF-κB pathway. For example, lncRNA CASC2, a tumor-suppressor lncRNA, was shown to impede the NF-κB pathway as miR-362-5p sponge, thereby hampering migration and invasion of HCC cells (73). By contrast, oncogenic lncRNAs that promote HCC development can serve as activators in NF-κB pathway *via* acting as ceRNAs by associating with miRNAs. For instance, lncRNA CRNDE, an oncogenic lncRNA, significantly enhanced phosphorylation of IκB by sponging miR-539-5p *via* a ceRNA-based mechanism, thereby promoting HCC progression (82). SNHG16, a widely studied tumor-associated lncRNA, which is often overexpressed in tumor tissues and mainly exerts a vital role in various malignant behaviors and events of tumors by sponging miRNAs. Generally, the higher the level of hepatic SNHG16, the worse the clinical situation (87). In HCC, SNHG16 was confirmed to promote tumor proliferation and metastasis by acting a “sponge” to absorb miR-17-5p, which in turn up-regulated p62, causing the downstream NF-κB signaling activation (83). Another study has pointed out that SNHG16 inhibits the activity of miR-605-3p as a ceRNA, which in turn restored the expression of TRAF6 and went against HCC mitigation (56). TP73-AS1 was an oncogenic lncRNA that targeted miR-200a to reduce its inhibiting effect on HMGB1, which promoted NF-κB signaling of HCC and its downstream cytokines levels (84). Similarly, activation of NF-κB signaling was involved in SNHG12-mediated hepatocarcinogenesis. The generation of SNHG12 was essential for sponging more miR-199a/b-5p molecules, which resulted in the upregulation of MLK3 that functioned as an IκB kinase kinase (IKKK) (88). Additionally, a study proved that NEAT1 could act as a ceRNA to regulate miR-129-5p availability for its target gene, VCP and IκB, and thus promoting the proliferation of HCC cells (89).

### LncRNAs Involved in the Regulation of NF-κB Pathway by Interacting With Proteins

In addition to regulating transcription and functioning as miRNA sponges, lncRNAs can also modulate NF-κB signaling in HCC by mediating protein degradation and stabilization. For instance, lncRNA miR503HG mediated HNRNPA2B1 degradation by means of the ubiquitin-proteasome pathway, thus reducing transcription of p52 and p65 in HCC cells (78). In contrast, LINC00665 maintained the protein stability of PKR by interdicting its degradation, thereby mediating NF-κB signaling activation in HCC (90). Moreover, NKILA was reported to bind to the NF-κB/IκB complex in such a way as to mask the phosphorylation of IκB, thus contributing to protein complex stability, causing a negative feedback loop of NF-κB pathway in HCC (80).

## NF-κB-Associated Chemoresistance in HCC and ncRNAs-Targeting Therapy

With the emergence of new drugs and the standardization of chemotherapy regimens, chemotherapy has become one of the most important modes of cancer treatment besides surgery, which has improved the survival rate and time of tumor patients to a certain extent. However, chemotherapy has its own limitations, such as high toxicity, immunosuppression, and primary and/or secondary resistance of tumor cells(chemoresistance). Of the three limitations listed, chemoresistance poses the greatest obstacle to the effective treatment of HCC patients using chemotherapy (91). Therefore, in order to improve the efficacy of chemotherapy and overcome chemoresistance, the mechanisms of chemoresistance and the molecular regulatory networks implicated in HCC still need to be further studied. It has been detected that NF-κB signaling is frequently activated in HCC, which is closely related to the onset of chemoresistance in this setting (92, 93). In addition to the induction of NF-κB signaling by extracellular ligand/cell-surface receptors interactions, chemotherapy-induced DNA damage can also activate NF-κB, leading to the transcription of numerous NF-κB-activated anti-apoptotic genes, the desensitization of cells to apoptosis, and further promotion of cancer progression (94, 95). Over the past years, the regulatory function of ncRNAs in hepatocarcinogenesis and chemoresistance has attracted extensive attention (10). As some reports show promising data, targeting the NF-κB pathway by ncRNAs seems to improve chemosensitivity of patients with HCC to chemotherapeutic agents.

Paclitaxel is one of the most widely used chemotherapy drugs, employed in the treatment of various malignant tumors (96–98), including HCC. However, chemoresistance of paclitaxel often occurs in patients with HCC, with NF-κB signaling being implicated in the mechanisms of paclitaxel-specific chemoresistance. As mentioned above, ncRNAs are of great use in improving the efficacy and chemosensitivity by targeting NF-κB signaling. For example, knocking down the expression of miR-16 increased the chemoresistance of HCC cell lines to paclitaxel through the NF-κB signaling, and the restoration of miR-16 expression effectively reversed chemoresistance of HCC by targeting IKKβ (30). Doxorubicin, another chemotherapeutic agent widely used against various malignant tumors, mainly interferes with the function of DNA topoisomerase II-α and breaks DNA double-strand to induce apoptosis of tumor cells (99). In HCC, chemoresistance to doxorubicin is another clinical problem yet to be solved. Doxorubicin was found to dramatically elevate phosphorylation level of p65, leading to the activation of numerous anti-apoptotic genes in HCC cells. However, restoring the expression of miR-26b dramatically blocked the nuclear translocation of NF-κB, further decreasing the occurrence of NF-κB-mediated chemoresistance of HCC cells to doxorubicin (29). Another study has proved that the over-expression of lncRNA 00607 enhances the sensitization of HCC cells to doxorubicin and other chemotherapeutic drugs *via* NF-κB p65/p53 signaling axis (81). LncRNA PDIA3P1 was an oncogenic lncRNAs and its presence was also associated with chemoresistance of HCC to doxorubicin, which protected HCC cells from doxorubicin-induced apoptosis through NF-κB activation. Therefore, inhibition of PDIA3P1 was a useful method to restore the chemosensitivity of HCC to doxorubicin (55). Cisplatin is a platinum-containing anticancer drug, which functions to facilitate the apoptosis of cancer cells through the interference in DNA repair mechanisms and the induction of DNA damage (100). Recently, a study uncovered that the chemosensitivity of HCC to cisplatin was correlated with the dysregulation of miR-1180. The higher the expression of miR-1180, the more severe the extent of chemoresistance. In terms of mechanism, high expression of miR-1180 facilitated the downregulation of NF-κB-negative regulators which in turn caused the NF-κB-mediated chemoresistance of HCC cells to cisplatin (50). Sorafenib is a first-line chemotherapy agent for advanced HCC patients (101). As an oral multikinase inhibitor, sorafenib plays an anti-cancer role by inhibiting cell proliferation and angiogenesis (102). Unfortunately, most HCC patients are prone to develop chemoresistance to sorafenib during treatment and ultimately gain poor clinical outcomes (102). Given the central role of sorafenib in HCC therapy, it is urgent to further study the exact mechanisms of sorafenib resistance in HCC so as to improve chemosensitivity of HCC to sorafenib. Recent studies have revealed that the activation of NF‐κB is identified as a crucial molecule leading to sorafenib resistance in HCC (102–104). Meanwhile, ncRNAs have been considered to be vital regulators in sorafenib resistance of HCC (105). Notably, certain ncRNAs that are discussed in this review have been confirmed to be involved in sorafenib resistance in HCC, such as miR-124, NEAT1, SNHG16 and so on. Therefore, subsequent studies will need to focus on understanding whether these ncRNAs are implicated in the development of NF‐κB-mediated sorafenib resistance in HCC.

## Perspectives

HCC can be in part ascribed to NF-κB signaling activation, the aberrant activation of which is linked with initiation, progression, metastasis, and drug resistance of HCC. As discussed in this review, small and long ncRNAs have emerged as promising molecules for regulating NF-κB signaling, and the restoration or inhibition of ncRNAs expression levels has shown high therapeutic potential in HCC. Generally, there are two therapeutic strategies that target ncRNAs in HCC. The first method aims to restore the tumor suppressor activity of ncRNAs that are lost or downregulated in HCC by using synthetic ncRNA molecules with same function, such as ncRNA mimics or ncRNAs expression vectors. The second approach aims to block the oncogenic activity of ncRNAs that are abnormally overexpressed in HCC. Both strategies can be applied to miRNAs, but in the case of lncRNAs, blocking their function is more reasonable than restoring the biological activity of these transcripts (106). CircRNAs have become a latest research hotspot in the field of ncRNAs, and their potential clinical value has been widely studied. Compared with linear RNAs, circRNAs are highly stable and covalently closed loop transcripts without 5′ caps and 3′ tails (107, 108). They are widely found in a variety of eukaryotes with extremely significant biological functions (109). With the development of high-throughput sequencing techniques, numerous circRNAs have been discovered to be correlated with the occurrence and development of various diseases, which especially exert an important influence on the pathogenesis, diagnosis, treatment and prognosis of tumors (109, 110). Recent evidence indicates that circRNAs might involve in the pathogenesis of HCC and exert their regulatory roles in HCC mainly by sponging miRNAs (109). Although there have been few reports on the crosstalk between circRNAs and NF-κB in HCC, some circRNAs have been discovered to be able to sponge the miRNAs that are reported herein which can mediate NF-κB signaling in HCC. For example, circZNF609-miR-342-3p (111), circPTGR-miR-129-5p (112) and circHIPK3-miR-124 (113) pathways have been discovered in HCC recently, suggesting that these circRNAs that function as ceRNAs might mediate HCC progression though regulating NF-κB signaling. Moreover, circRNAs have also been discovered to have complex roles in mediating NF-κB signaling, which contributes to the development of colorectal cancer (114), ovarian cancer (115), breast cancer (116) and other cancers (117, 118). This evidence further confirms that circRNA-NF-κB pathway may serve as a novel future research direction in HCC.

Although ncRNAs represent promising targets for human cancer therapeutic interventions, several issues still need to be addressed. First, the relationship between the dysregulation of ncRNAs and HCC remains unclear based on the current studies, further in-depth research is needed to ascertain whether the dysregulation of ncRNAs leads to the occurrence and development of HCC or whether the development of HCC causes the abnormal expression of ncRNAs in the first place. Second, the tumor microenvironment of HCC is a complex system composed of many cell subsets. It will be quite crucial to elucidate the cellular sources of the abnormally expressed ncRNAs in the tumor microenvironment of HCC, and define the mechanisms employed by those ncRNAs in the initiation and development of HCC. Future research will need to focus on understanding the origins, action targets, traits and functions of ncRNAs, particularly identifying the characteristics of deterministic ncRNAs in HCC and the specific action targets of these ncRNAs through large-scale and comprehensive analytical studies. These findings will help to develop potential diagnostic, therapeutic, and prognostic approaches for HCC. Finally, the development of ncRNA-based anti-HCC therapies is still in its infancy, therefore, more attention should be paid to the multi-targets, off-target, instability and other defects in the research and clinical application of ncRNA mimics and antagonists, so as to strengthen anti-HCC therapeutic efficacy and reduce side effects.

## Author Contributions

YZ wrote the manuscript. JS prepared figures. SL made tables. MZ and YL determined the topic of the manuscript and participated in its coordination and modification. All authors contributed to the article and approved the submitted version.

## Funding

The study was supported by Grants from the National Nature Science Foundation of China, No. U20A20348, the National Nature Science Foundation of China, No. 81871646, the Scientific and Technological Innovation Leading Talents of “Ten Thousand Talents Plan” of Zhejiang Province, No. 2020R52010.

## Conflict of Interest

The authors declare that the research was conducted in the absence of any commercial or financial relationships that could be construed as a potential conflict of interest.

## Publisher’s Note

All claims expressed in this article are solely those of the authors and do not necessarily represent those of their affiliated organizations, or those of the publisher, the editors and the reviewers. Any product that may be evaluated in this article, or claim that may be made by its manufacturer, is not guaranteed or endorsed by the publisher.

## Abbreviations

HCC, Hepatocellular carcinoma; NF-κB, Nuclear factor kappa B; ncRNA, non-coding RNA; miRNA, microRNA; lncRNA, long non-coding RNA; IκB, Inhibitor of κB; IKK, IκB kinase; NEMO, NF-κB essential modulator; TNFR, Tumour necrosis factor receptor; NIK, NF-κB-inducing kinase; piRNA, PIWI interacting RNA; siRNA, small interfering RNA; circRNA, circular RNA; mRNA, messenger RNA; UTR, Untranslated region; TRAF, Tumor necrosis factor receptor-associated factor; TAK1, Transforming growth factor-β-activated kinase 1; TAB, TAK-binding protein; BLVRB, Biliverdin reductase B; TRIM23, Transcripts encoding tripartite motif containing 23; Lin28A, Lin28 homolog A; VCP, Valosin containing protein; MAP4K4, Mitogen-activated protein 4 kinase 4; Dnmt1, DNA methyltransferase 1; BIRC3, Baculoviral IAP Repeat Containing 3; ROCK1, Rho-associated protein kinase 1; TLE1, Transducin-like Enhancer Protein 1; IL-1R/TLR, Interleukin-1 receptor/Toll-like receptor; MAPKKK/MAP3K, Mitogen-activated protein kinase kinase kinaser; DUB, Deubiquitinating enzyme; STAU1, Staufen 1; HNRNPA2B1, Heterogeneous nuclear ribonucleoprotein A2/B1; NKILA, NF-κB interacting lncRNA; CASC2, Cancer susceptibility candidate 2; PDIA3P1, Protein disulfide isomerase family A member 3 pseudogene 1; CRNDE, Colorectal Neoplasia Differentially Expressed; SNHG16, Small nucleolar RNA host gene 16; TP73-AS1, P73 antisense RNA 1 T; N/A, Not available; SNHG12, Small nucleolar RNA host gene 12; PKR, Double-stranded RNA (dsRNA)-activated protein kinase; NEAT1, Nuclear-enriched abundant transcript 1; ceRNA, competitive endogenous RNA; IKKK, IκB kinase kinase.

## References

[1] Durand JK Baldwin AS. Targeting IKK and NF-κB for Therapy. Adv Protein Chem Struct Biol (2017) 107:77–115. doi: 10.1016/bs.apcsb.2016.11.006 28215229

[2] Kim HR Lee SH Jung G. The Hepatitis B Viral X Protein Activates NF-kappaB Signaling Pathway Through the Up-Regulation of TBK1. FEBS Lett (2010) 584:525–30. doi: 10.1016/j.febslet.2009.11.091 19958770

[3] Tai DI Tsai SL Chen YM Chuang YL Peng CY Sheen IS. Activation of Nuclear Factor kappaB in Hepatitis C Virus Infection: Implications for Pathogenesis and Hepatocarcinogenesis. Hepatology (2000) 31:656–64. doi: 10.1002/hep.510310316 10706556

[4] Arsura M Cavin LG. Nuclear factor-kappaB and Liver Carcinogenesis. Cancer Lett (2005) 229:157–69. doi: 10.1016/j.canlet.2005.07.008 16125305

[5] Luedde T Schwabe RF. NF-κB in the Liver–Linking Injury, Fibrosis and Hepatocellular Carcinoma. Nat Rev Gastroenterol Hepatol (2011) 8:108–18. doi: 10.1038/nrgastro.2010.213 PMC3295539 21293511

[6] Pikarsky E Porat RM Stein I Abramovitch R Amit S Kasem S. NF-kappaB Functions as a Tumour Promoter in Inflammation-Associated Cancer. Nature (2004) 431:461–6. doi: 10.1038/nature02924 15329734

[7] Pan H Fu X Huang W. Molecular Mechanism of Liver Cancer. Anticancer Agents Med Chem (2011) 11:493–9. doi: 10.2174/187152011796011073 21554201

[8] Llovet JM Zucman-Rossi J Pikarsky E Sangro B Schwartz M Sherman M. Hepatocellular Carcinoma. Nat Rev Dis Primers (2016) 2:16018. doi: 10.1038/nrdp.2016.18 27158749

[9] Sahu SK Chawla YK Dhiman RK Singh V Duseja A Taneja S. Rupture of Hepatocellular Carcinoma: A Review of Literature. J Clin Exp Hepatol (2019) 9:245–56. doi: 10.1016/j.jceh.2018.04.002 PMC6476943 31024207

[10] Wei L Wang X Lv L Liu J Xing H Song Y. The Emerging Role of microRNAs and Long Noncoding RNAs in Drug Resistance of Hepatocellular Carcinoma. Mol Cancer (2019) 18:147. doi: 10.1186/s12943-019-1086-z 31651347 PMC6814027

[11] Smale ST. Dimer-Specific Regulatory Mechanisms Within the NF-κB Family of Transcription Factors. Immunol Rev (2012) 246:193–204. doi: 10.1111/j.1600-065X.2011.01091.x 22435556

[12] Ghosh S May MJ Kopp EB. NF-Kappa B and Rel Proteins: Evolutionarily Conserved Mediators of Immune Responses. Annu Rev Immunol (1998) 16:225–60. doi: 10.1146/annurev.immunol.16.1.225 9597130

[13] Mitchell S Vargas J Hoffmann A. Signaling *via* the NF-κB System. Wiley Interdiscip Rev Syst Biol Med (2016) 8:227–41. doi: 10.1002/wsbm.1331 PMC8363188 26990581

[14] Bhoj VG Chen ZJ. Ubiquitylation in Innate and Adaptive Immunity. Nature (2009) 458:430–7. doi: 10.1038/nature07959 19325622

[15] Yaron A Hatzubai A Davis M Lavon I Amit S Manning AM. Identification of the Receptor Component of the IkappaBalpha-Ubiquitin Ligase. Nature (1998) 396:590–4. doi: 10.1038/25159 9859996

[16] Hayden MS Ghosh S. Signaling to NF-kappaB. Genes Dev (2004) 18:2195–224. doi: 10.1101/gad.1228704 15371334

[17] Mulero MC Huxford T Ghosh G. NF-κB, IκB, and IKK: Integral Components of Immune System Signaling. Adv Exp Med Biol (2019) 1172:207–26. doi: 10.1007/978-981-13-9367-9_10 31628658

[18] Hombach S Kretz M. Non-Coding RNAs: Classification, Biology and Functioning. Adv Exp Med Biol (2016) 937:3–17. doi: 10.1007/978-3-319-42059-2_1 27573892

[19] Zhang P Wu W Chen Q Chen M. Non-Coding RNAs and Their Integrated Networks. J Integr Bioinform (2019) 16:1–12. doi: 10.1515/jib-2019-0027 PMC6798851 31301674

[20] Negrini M Nicoloso MS Calin GA. MicroRNAs and Cancer–New Paradigms in Molecular Oncology. Curr Opin Cell Biol (2009) 21:470–9. doi: 10.1016/j.ceb.2009.03.002 19411171

[21] Di Leva G Garofalo M Croce CM. MicroRNAs in Cancer. Annu Rev Pathol (2014) 9:287–314. doi: 10.1146/annurev-pathol-012513-104715 24079833 PMC4009396

[22] Hayes J Peruzzi PP Lawler S. MicroRNAs in Cancer: Biomarkers, Functions and Therapy. Trends Mol Med (2014) 20:460–9. doi: 10.1016/j.molmed.2014.06.005 25027972

[23] Reddy KB. MicroRNA (miRNA) in Cancer. Cancer Cell Int (2015) 15:38. doi: 10.1186/s12935-015-0185-1 25960691 PMC4424445

[24] Huang S He X. The Role of microRNAs in Liver Cancer Progression. Br J Cancer (2011) 104:235–40. doi: 10.1038/sj.bjc.6606010 PMC3031886 21102580

[25] Law PT Wong N. Emerging Roles of microRNA in the Intracellular Signaling Networks of Hepatocellular Carcinoma. J Gastroenterol Hepatol (2011) 26:437–49. doi: 10.1111/j.1440-1746.2010.06512.x 21332540

[26] Wang P Cao J Liu S Pan H Liu X Sui A. Upregulated microRNA-429 Inhibits the Migration of HCC Cells by Targeting TRAF6 Through the NF-κB Pathway. Oncol Rep (2017) 37:2883–90. doi: 10.3892/or.2017.5507 28440423

[27] Fei X Zhang P Pan Y Liu Y. MicroRNA-98-5p Inhibits Tumorigenesis of Hepatitis B Virus-Related Hepatocellular Carcinoma by Targeting NF-κB-Inducing Kinase. Yonsei Med J (2020) 61:460–70. doi: 10.3349/ymj.2020.61.6.460 PMC7256008 32469170

[28] Zhang S Shan C Kong G Du Y Ye L Zhang X. MicroRNA-520e Suppresses Growth of Hepatoma Cells by Targeting the NF-κB-Inducing Kinase (NIK). Oncogene (2012) 31:3607–20. doi: 10.1038/onc.2011.523 22105365

[29] Zhao N Wang R Zhou L Zhu Y Gong J Zhuang SM. MicroRNA-26b Suppresses the NF-κB Signaling and Enhances the Chemosensitivity of Hepatocellular Carcinoma Cells by Targeting TAK1 and TAB3. Mol Cancer (2014) 13:35. doi: 10.1186/1476-4598-13-35 24565101 PMC3938074

[30] Huang Y Chen G Wang Y He R Du J Jiao X. Inhibition of microRNA-16 Facilitates the Paclitaxel Resistance by Targeting IKBKB *via* NF-κB Signaling Pathway in Hepatocellular Carcinoma. Biochem Biophys Res Commun (2018) 503:1035–41. doi: 10.1016/j.bbrc.2018.06.113 29935185

[31] Li HP Zeng XC Zhang B Long JT Zhou B Tan GS. MiR-451 Inhibits Cell Proliferation in Human Hepatocellular Carcinoma Through Direct Suppression of IKK-β. Carcinogenesis (2013) 34:2443–51. doi: 10.1093/carcin/bgt206 23740840

[32] Ding J Huang S Wang Y Tian Q Zha R Shi H. Genome-Wide Screening Reveals That miR-195 Targets the TNF-α/NF-κB Pathway by Down-Regulating IκB Kinase Alpha and TAB3 in Hepatocellular Carcinoma. Hepatology (2013) 58:654–66. doi: 10.1002/hep.26378 23487264

[33] Zhao L Zhang Y. MiR-342-3p Affects Hepatocellular Carcinoma Cell Proliferation *via* Regulating NF-κB Pathway. Biochem Biophys Res Commun (2015) 457:370–7. doi: 10.1016/j.bbrc.2014.12.119 25580008

[34] Huan L Bao C Chen D Li Y Lian J Ding J. MicroRNA-127-5p Targets the Biliverdin Reductase B/nuclear Factor-κB Pathway to Suppress Cell Growth in Hepatocellular Carcinoma Cells. Cancer Sci (2016) 107:258–66. doi: 10.1111/cas.12869 PMC4814244 26708147

[35] Su XF Li N Meng FL Chu YL Li T Gao XZ. MiR-16 Inhibits Hepatocellular Carcinoma Progression by Targeting FEAT Through NF-κB Signaling Pathway. Eur Rev Med Pharmacol Sci (2019) 23:10274–82. doi: 10.26355/eurrev_201912_19665 31841182

[36] Bao C Li Y Huan L Zhang Y Zhao F Wang Q. NF-κB Signaling Relieves Negative Regulation by miR-194 in Hepatocellular Carcinoma by Suppressing the Transcription Factor HNF-1α. Sci Signal (2015) 8:a75. doi: 10.1126/scisignal.aaa8441 26221053

[37] Xu WP Yi M Li QQ Zhou WP Cong WM Yang Y. Perturbation of MicroRNA-370/Lin-28 Homolog A/nuclear Factor Kappa B Regulatory Circuit Contributes to the Development of Hepatocellular Carcinoma. Hepatology (2013) 58:1977–91. doi: 10.1002/hep.26541 23728999

[38] Liu Y Hei Y Shu Q Dong J Gao Y Fu H. VCP/p97, Down-Regulated by microRNA-129-5p, Could Regulate the Progression of Hepatocellular Carcinoma. PloS One (2012) 7:e35800. doi: 10.1371/journal.pone.0035800 22536440 PMC3335000

[39] Wang L Yao J Sun H Sun R Chang S Yang Y. MiR-302b Suppresses Cell Invasion and Metastasis by Directly Targeting AKT2 in Human Hepatocellular Carcinoma Cells. Tumour Biol (2016) 37:847–55. doi: 10.1007/s13277-015-3330-5 26254095

[40] Jiang F Wang X Liu Q Shen J Li Z Li Y. Inhibition of TGF-β/SMAD3/NF-κB Signaling by microRNA-491 is Involved in Arsenic Trioxide-Induced Anti-Angiogenesis in Hepatocellular Carcinoma Cells. Toxicol Lett (2014) 231:55–61. doi: 10.1016/j.toxlet.2014.08.024 25196641

[41] Song WH Feng XJ Gong SJ Chen JM Wang SM Xing DJ. MicroRNA-622 Acts as a Tumor Suppressor in Hepatocellular Carcinoma. Cancer Biol Ther (2015) 16:1754–63. doi: 10.1080/15384047.2015.1095402 PMC4847826 26467022

[42] Ma JH Bu X Wang JJ Xie YX. MicroRNA-29-3p Regulates Hepatocellular Carcinoma Progression Through NF-κB Pathway. Clin Lab (2019) 65:801–6. doi: 10.7754/Clin.Lab.2018.181012 31115237

[43] Wang H Jiang F Liu W Tian W. MiR-595 Suppresses Cell Proliferation and Metastasis in Hepatocellular Carcinoma by Inhibiting NF-κB Signalling Pathway. Pathol Res Pract (2020) 216:152899. doi: 10.1016/j.prp.2020.152899 32107085

[44] Takata A Otsuka M Yoshikawa T Kishikawa T Hikiba Y Obi S. MicroRNA-140 Acts as a Liver Tumor Suppressor by Controlling NF-κB Activity by Directly Targeting DNA Methyltransferase 1 (Dnmt1) Expression. Hepatology (2013) 57:162–70. doi: 10.1002/hep.26011 PMC3521841 22898998

[45] Cao J Qiu J Wang X Lu Z Wang D Feng H. Identification of microRNA-124 in Regulation of Hepatocellular Carcinoma Through BIRC3 and the NF-κB Pathway. J Cancer (2018) 9:3006–15. doi: 10.7150/jca.25956 PMC6134807 30210622

[46] Wang RK Shao XM Yang JP Yan HL Shao Y. MicroRNA-145 Inhibits Proliferation and Promotes Apoptosis of HepG2 Cells by Targeting ROCK1 Through the ROCK1/NF-κB Signaling Pathway. Eur Rev Med Pharmacol Sci (2019) 23:2777–85. doi: 10.26355/eurrev_201904_17551 31002128

[47] Zhou P Jiang W Wu L Chang R Wu K Wang Z. MiR-301a is a Candidate Oncogene That Targets the Homeobox Gene Gax in Human Hepatocellular Carcinoma. Dig Dis Sci (2012) 57:1171–80. doi: 10.1007/s10620-012-2099-2 22373864

[48] Lu S Wu J Gao Y Han G Ding W Huang X. MicroRNA-4262 Activates the NF-κB and Enhances the Proliferation of Hepatocellular Carcinoma Cells. Int J Biol Macromol (2016) 86:43–9. doi: 10.1016/j.ijbiomac.2016.01.019 26778158

[49] Zhang L Yang L Liu X Chen W Chang L Chen L. MicroRNA-657 Promotes Tumorigenesis in Hepatocellular Carcinoma by Targeting Transducin-Like Enhancer Protein 1 Through Nuclear Factor Kappa B Pathways. Hepatology (2013) 57:1919–30. doi: 10.1002/hep.26162 23175432

[50] Tan G Wu L Tan J Zhang B Tai WC Xiong S. MiR-1180 Promotes Apoptotic Resistance to Human Hepatocellular Carcinoma *via* Activation of NF-κB Signaling Pathway. Sci Rep (2016) 6:22328. doi: 10.1038/srep22328 26928365 PMC4772113

[51] Ni F Zhao H Cui H Wu Z Chen L Hu Z. MicroRNA-362-5p Promotes Tumor Growth and Metastasis by Targeting CYLD in Hepatocellular Carcinoma. Cancer Lett (2015) 356:809–18. doi: 10.1016/j.canlet.2014.10.041 25449782

[52] Chung JY Park YC Ye H Wu H. All TRAFs are Not Created Equal: Common and Distinct Molecular Mechanisms of TRAF-Mediated Signal Transduction. J Cell Sci (2002) 115:679–88. doi: 10.1242/jcs.115.4.679 11865024

[53] Deng L Wang C Spencer E Yang L Braun A You J. Activation of the IkappaB Kinase Complex by TRAF6 Requires a Dimeric Ubiquitin-Conjugating Enzyme Complex and a Unique Polyubiquitin Chain. Cell (2000) 103:351–61. doi: 10.1016/s0092-8674(00)00126-4 11057907

[54] Shi JH Sun SC. Tumor Necrosis Factor Receptor-Associated Factor Regulation of Nuclear Factor κB and Mitogen-Activated Protein Kinase Pathways. Front Immunol (2018) 9:1849. doi: 10.3389/fimmu.2018.01849 30140268 PMC6094638

[55] Xie C Zhang LZ Chen ZL Zhong WJ Fang JH Zhu Y. A Hmtr4-PDIA3P1-miR-125/124-TRAF6 Regulatory Axis and Its Function in NF Kappa B Signaling and Chemoresistance. Hepatology (2020) 71:1660–77. doi: 10.1002/hep.30931 PMC7318625 31509261

[56] Hu YL Feng Y Chen YY Liu JZ Su Y Li P. SNHG16/miR-605-3p/TRAF6/NF-κB Feedback Loop Regulates Hepatocellular Carcinoma Metastasis. J Cell Mol Med (2020) 24:7637–51. doi: 10.1111/jcmm.15399 PMC7339162 32436333

[57] Sun LL Wang J Zhao ZJ Liu N Wang AL Ren HY. Suppressive Role of miR-502-5p in Breast Cancer *via* Downregulation of TRAF2. Oncol Rep (2014) 31:2085–92. doi: 10.3892/or.2014.3105 24677135

[58] Ozata DM Li X Lee L Liu J Warsito D Hajeri P. Loss of miR-514a-3p Regulation of PEG3 Activates the NF-Kappa B Pathway in Human Testicular Germ Cell Tumors. Cell Death Dis (2017) 8:e2759. doi: 10.1038/cddis.2016.464 28471449 PMC5520681

[59] Jiang L Yu L Zhang X Lei F Wang L Liu X. miR-892b Silencing Activates NF-κB and Promotes Aggressiveness in Breast Cancer. Cancer Res (2016) 76:1101–11. doi: 10.1158/0008-5472.CAN-15-1770 26747895

[60] Sun SC. Non-Canonical NF-κb Signaling Pathway. Cell Res (2011) 21:71–85. doi: 10.1038/cr.2010.177 21173796 PMC3193406

[61] Malinin NL Boldin MP Kovalenko AV Wallach D. MAP3K-Related Kinase Involved in NF-κB Induction by TNF, CD95 and IL-1. Nature (1997) 385:540–4. doi: 10.1038/385540a0 9020361

[62] Maubach G Feige MH Lim M Naumann M. NF-kappaB-Inducing Kinase in Cancer. Biochim Biophys Acta Rev Cancer (2019) 1871:40–9. doi: 10.1016/j.bbcan.2018.10.002 30419317

[63] Yamaguchi K Shirakabe K Shibuya H Irie K Oishi I Ueno N. Identification of a Member of the MAPKKK Family as a Potential Mediator of TGF-Beta Signal Transduction. Science (1995) 270:2008–11. doi: 10.1126/science.270.5244.2008 8533096

[64] Ninomiya-Tsuji J Kishimoto K Hiyama A Inoue J Cao Z Matsumoto K. The Kinase TAK1 can Activate the NIK-I kappaB as Well as the MAP Kinase Cascade in the IL-1 Signalling Pathway. Nature (1999) 398:252–6. doi: 10.1038/18465 10094049

[65] Xiao F Wang H Fu X Li Y Wu Z. TRAF6 Promotes Myogenic Differentiation *via* the TAK1/p38 Mitogen-Activated Protein Kinase and Akt Pathways. PloS One (2012) 7:e34081. doi: 10.1371/journal.pone.0034081 22496778 PMC3319550

[66] Chen ZJ. Ubiquitination in Signaling to and Activation of IKK. Immunol Rev (2012) 246:95–106. doi: 10.1111/j.1600-065X.2012.01108.x 22435549 PMC3549672

[67] Huang JY Zhang K Chen DQ Chen J Feng B Song H. MicroRNA-451: Epithelial-Mesenchymal Transition Inhibitor and Prognostic Biomarker of Hepatocelluar Carcinoma. Oncotarget (2015) 6:18613–30. doi: 10.18632/oncotarget.4317 PMC4621914 26164082

[68] Liu X Zhang A Xiang J Lv Y Zhang X. miR-451 Acts as a Suppressor of Angiogenesis in Hepatocellular Carcinoma by Targeting the IL-6r-STAT3 Pathway. Oncol Rep (2016) 36:1385–92. doi: 10.3892/or.2016.4971 27461244

[69] He JF Luo YM Wan XH Jiang D. Biogenesis of MiRNA-195 and its Role in Biogenesis, the Cell Cycle, and Apoptosis. J Biochem Mol Toxicol (2011) 25:404–8. doi: 10.1002/jbt.20396 22190509

[70] Lork M Verhelst K Beyaert R. CYLD, A20 and OTULIN Deubiquitinases in NF-κB Signaling and Cell Death: So Similar, Yet So Different. Cell Death Differ (2017) 24:1172–83. doi: 10.1038/cdd.2017.46 PMC5520167 28362430

[71] Hu H Brittain GC Chang JH Puebla-Osorio N Jin J Zal A. OTUD7B Controls Non-Canonical NF-κB Activation Through Deubiquitination of TRAF3. Nature (2013) 494:371–4. doi: 10.1038/nature11831 PMC3578967 23334419

[72] Van Huffel S Delaei F Heyninck K De Valck D Beyaert R. Identification of a Novel A20-Binding Inhibitor of Nuclear Factor-Kappa B Activation Termed ABIN-2. J Biol Chem (2001) 276:30216–23. doi: 10.1074/jbc.M100048200 11390377

[73] Zhao L Zhang Y Zhang Y. Long Noncoding RNA CASC2 Regulates Hepatocellular Carcinoma Cell Oncogenesis Through miR-362-5p/NF-κB Axis. J Cell Physiol (2018) 233:6661–70. doi: 10.1002/jcp.26446 29319182

[74] Dai RM Chen E Longo DL Gorbea CM Li CC. Involvement of Valosin-Containing Protein, an ATPase Co-Purified With IkappaBalpha and 26 S Proteasome, in Ubiquitin-Proteasome-Mediated Degradation of IkappaBalpha. J Biol Chem (1998) 273:3562–73. doi: 10.1074/jbc.273.6.3562 9452483

[75] Dai RM Li CC. Valosin-Containing Protein Is a Multi-Ubiquitin Chain-Targeting Factor Required in Ubiquitin-Proteasome Degradation. Nat Cell Biol (2001) 3:740–4. doi: 10.1038/35087056 11483959

[76] Fatica A Bozzoni I. Long Non-Coding RNAs: New Players in Cell Differentiation and Development. Nat Rev Genet (2014) 15:7–21. doi: 10.1038/nrg3606 24296535

[77] Huang Z Zhou JK Peng Y He W Huang C. The Role of Long Noncoding RNAs in Hepatocellular Carcinoma. Mol Cancer (2020) 19:77. doi: 10.1186/s12943-020-01188-4 32295598 PMC7161154

[78] Wang H Liang L Dong Q Huan L He J Li B. Long Noncoding RNA Mir503hg, a Prognostic Indicator, Inhibits Tumor Metastasis by Regulating the HNRNPA2B1/NF-κB Pathway in Hepatocellular Carcinoma. Theranostics (2018) 8:2814–29. doi: 10.7150/thno.23012 PMC5957011 29774077

[79] Ke S Li RC Meng FK Fang MH. NKILA Inhibits NF-κB Signaling and Suppresses Tumor Metastasis. Aging (Albany NY) (2018) 10:56–71. doi: 10.18632/aging.101359 29348395 PMC5811242

[80] Chen R Cheng Q Owusu-Ansah KG Song G Jiang D Zhou L. NKILA, a Prognostic Indicator, Inhibits Tumor Metastasis by Suppressing NF-κB/Slug Mediated Epithelial-Mesenchymal Transition in Hepatocellular Carcinoma. Int J Biol Sci (2020) 16:495–503. doi: 10.7150/ijbs.39582 32015685 PMC6990899

[81] Sun QM Hu B Fu PY Tang WG Zhang X Zhan H. Long Non-Coding RNA 00607 as a Tumor Suppressor by Modulating NF-κB P65/P53 Signaling Axis in Hepatocellular Carcinoma. Carcinogenesis (2018) 39:1438–46. doi: 10.1093/carcin/bgy113 30169594

[82] Li Z Wu G Li J Wang Y Ju X Jiang W. LncRNA CRNDE Promotes the Proliferation and Metastasis by Acting as Sponge miR-539-5p to Regulate POU2F1 Expression in HCC. BMC Cancer (2020) 20:282. doi: 10.1186/s12885-020-06771-y 32252678 PMC7137470

[83] Zhong JH Xiang X Wang YY Liu X Qi LN Luo CP. The lncRNA SNHG16 Affects Prognosis in Hepatocellular Carcinoma by Regulating P62 Expression. J Cell Physiol (2020) 235:1090–102. doi: 10.1002/jcp.29023 31256427

[84] Li S Huang Y Huang Y Fu Y Tang D Kang R. The Long Non-Coding RNA TP73-AS1 Modulates HCC Cell Proliferation Through miR-200a-Dependent HMGB1/RAGE Regulation. J Exp Clin Cancer Res (2017) 36:51. doi: 10.1186/s13046-017-0519-z 28403886 PMC5389141

[85] Xu X Yin Y Tang J Xie Y Han Z Zhang X. Long Non-Coding RNA Myd88 Promotes Growth and Metastasis in Hepatocellular Carcinoma *via* Regulating Myd88 Expression Through H3K27 Modification. Cell Death Dis (2017) 8:e3124. doi: 10.1038/cddis.2017.519 29022910 PMC5682683

[86] Salmena L Poliseno L Tay Y Kats L. Pandolfi PP. A ceRNA Hypothesis: The Rosetta Stone of a Hidden RNA Language? Cell (2011) 146:353–8. doi: 10.1016/j.cell.2011.07.014 PMC3235919 21802130

[87] Yang M Wei W. SNHG16: A Novel Long-Non Coding RNA in Human Cancers. Onco Targets Ther (2019) 12:11679–90. doi: 10.2147/OTT.S231630 PMC6942535 32021246

[88] Lan T Ma W Hong Z Wu L Chen X Yuan Y. Long Non-Coding RNA Small Nucleolar RNA Host Gene 12 (SNHG12) Promotes Tumorigenesis and Metastasis by Targeting miR-199a/B-5p in Hepatocellular Carcinoma. J Exp Clin Cancer Res (2017) 36:11. doi: 10.1186/s13046-016-0486-9 28073380 PMC5223416

[89] Fang L Sun J Pan Z Song Y Zhong L Zhang Y. Long Non-Coding RNA NEAT1 Promotes Hepatocellular Carcinoma Cell Proliferation Through the Regulation of miR-129-5p-VCP-IκB. Am J Physiol Gastrointest Liver Physiol (2017) 313:G150–6. doi: 10.1152/ajpgi.00426.2016 28526689

[90] Ding J Zhao J Huan L Liu Y Qiao Y Wang Z. Inflammation-Induced Long Intergenic Noncoding RNA (LINC00665) Increases Malignancy Through Activating the Double-Stranded RNA-Activated Protein Kinase/Nuclear Factor Kappa B Pathway in Hepatocellular Carcinoma. Hepatology (2020) 72:1666–81. doi: 10.1002/hep.31195 32083756

[91] Zheng HC. The Molecular Mechanisms of Chemoresistance in Cancers. Oncotarget (2017) 8:59950–64. doi: 10.18632/oncotarget.19048 PMC5601792 28938696

[92] Wu W Yang JL Wang YL Wang H Yao M Wang L. Reversal of Multidrug Resistance of Hepatocellular Carcinoma Cells by Metformin Through Inhibiting NF-κB Gene Transcription. World J Hepatol (2016) 8:985–93. doi: 10.4254/wjh.v8.i23.985 PMC4990762 27621764

[93] Ma W Sze KM Chan LK Lee JM Wei LL Wong CM. RhoE/ROCK2 Regulates Chemoresistance Through NF-κB /IL-6/ STAT3 Signaling in Hepatocellular Carcinoma. Oncotarget (2016) 7:41445–59. doi: 10.18632/oncotarget.9441 PMC5173071 27213590

[94] McCool KW Miyamoto S. DNA Damage-Dependent NF-κB Activation: NEMO Turns Nuclear Signaling Inside Out. Immunol Rev (2012) 246:311–26. doi: 10.1111/j.1600-065X.2012.01101.x PMC3311051 22435563

[95] Perkins ND. The Diverse and Complex Roles of NF-κB Subunits in Cancer. Nat Rev Cancer (2012) 12:121–32. doi: 10.1038/nrc3204 22257950

[96] Henley D Isbill M Fernando R Foster JS Wimalasena J. Paclitaxel Induced Apoptosis in Breast Cancer Cells Requires Cell Cycle Transit But Not Cdc2 Activity. Cancer Chemother Pharmacol (2007) 59:235–49. doi: 10.1007/s00280-006-0262-1 16972069

[97] Ofir R Seidman R Rabinski T Krup M Yavelsky V Weinstein Y. Taxol-Induced Apoptosis in Human SKOV3 Ovarian and MCF7 Breast Carcinoma Cells is Caspase-3 and Caspase-9 Independent. Cell Death Differ (2002) 9:636–42. doi: 10.1038/sj.cdd.4401012 12032672

[98] Frankel A Buckman R Kerbel RS. Abrogation of Taxol-Induced G2-M Arrest and Apoptosis in Human Ovarian Cancer Cells Grown as Multicellular Tumor Spheroids. Cancer Res (1997) 57:2388–93. 9192815

[99] Tacar O Sriamornsak P Dass CR. Doxorubicin: An Update on Anticancer Molecular Action, Toxicity and Novel Drug Delivery Systems. J Pharm Pharmacol (2013) 65:157–70. doi: 10.1111/j.2042-7158.2012.01567.x 23278683

[100] Dasari S Tchounwou PB. Cisplatin in Cancer Therapy: Molecular Mechanisms of Action. Eur J Pharmacol (2014) 740:364–78. doi: 10.1016/j.ejphar.2014.07.025 PMC4146684 25058905

[101] Cheng AL Kang YK Chen Z Tsao CJ Qin S Kim JS. Efficacy and Safety of Sorafenib in Patients in the Asia-Pacific Region With Advanced Hepatocellular Carcinoma: A Phase III Randomised, Double-Blind, Placebo-Controlled Trial. Lancet Oncol (2009) 10:25–34. doi: 10.1016/S1470-2045(08)70285-7 19095497

[102] Yu J Wang N Gong Z Liu L Yang S Chen GG. Cytochrome P450 1A2 Overcomes Nuclear Factor Kappa B-Mediated Sorafenib Resistance in Hepatocellular Carcinoma. Oncogene (2021) 40:492–507. doi: 10.1038/s41388-020-01545-z 33184472

[103] Liu J Liu Y Meng L Liu K Ji B. Targeting the PD-L1/DNMT1 Axis in Acquired Resistance to Sorafenib in Human Hepatocellular Carcinoma. Oncol Rep (2017) 38:899–907. doi: 10.3892/or.2017.5722 28627705 PMC5561980

[104] Gao L Morine Y Yamada S Saito Y Ikemoto T Tokuda K. The BAFF/NF-κB Axis is Crucial to Interactions Between Sorafenib-Resistant HCC Cells and Cancer-Associated Fibroblasts. Cancer Sci (2021) 112:3545–54. doi: 10.1111/cas.15041 PMC8409310 34159680

[105] Hu X Zhu H Shen Y Zhang X He X Xu X. The Role of Non-Coding RNAs in the Sorafenib Resistance of Hepatocellular Carcinoma. Front Oncol (2021) 11:696705. doi: 10.3389/fonc.2021.696705 34367979 PMC8340683

[106] Ling H. Non-Coding RNAs: Therapeutic Strategies and Delivery Systems. Adv Exp Med Biol (2016) 937:229–37. doi: 10.1007/978-3-319-42059-2_12 27573903

[107] Chen LL. The Expanding Regulatory Mechanisms and Cellular Functions of Circular RNAs. Nat Rev Mol Cell Biol (2020) 21:475–90. doi: 10.1038/s41580-020-0243-y 32366901

[108] Kristensen LS Andersen MS Stagsted L Ebbesen KK Hansen TB Kjems J. The Biogenesis, Biology and Characterization of Circular RNAs. Nat Rev Genet (2019) 20:675–91. doi: 10.1038/s41576-019-0158-7 31395983

[109] Shen H Liu B Xu J Zhang B Wang Y Shi L. Circular RNAs: Characteristics, Biogenesis, Mechanisms and Functions in Liver Cancer. J Hematol Oncol (2021) 14:134. doi: 10.1186/s13045-021-01145-8 34461958 PMC8407006

[110] Du WW Zhang C Yang W Yong T Awan FM Yang BB. Identifying and Characterizing circRNA-Protein Interaction. Theranostics (2017) 7:4183–91. doi: 10.7150/thno.21299 PMC5695005 29158818

[111] Liao X Zhan W Tian B Luo Y Gu F Li R. Circular RNA ZNF609 Promoted Hepatocellular Carcinoma Progression by Upregulating PAP2C Expression *via* Sponging miR-342-3p. Onco Targets Ther (2020) 13:7773–83. doi: 10.2147/OTT.S253936 PMC7414977 32801783

[112] Li X Zhang T. Circular RNA PTGR1 Regulates 5-FU Resistance and Development of Hepatocellular Carcinoma Cells by Modulating miR-129-5p/ABCC1 Axis. Cell Biol Int (2021) 45:2391. doi: 10.1002/cbin.11635 34003528

[113] Chen G Shi Y Liu M Sun J. Circhipk3 Regulates Cell Proliferation and Migration by Sponging miR-124 and Regulating AQP3 Expression in Hepatocellular Carcinoma. Cell Death Dis (2018) 9:175. doi: 10.1038/s41419-017-0204-3 29415990 PMC5833724

[114] Chen J Yang X Liu R Wen C Wang H Huang L. Circular RNA GLIS2 Promotes Colorectal Cancer Cell Motility *via* Activation of the NF-κB Pathway. Cell Death Dis (2020) 11:788. doi: 10.1038/s41419-020-02989-7 32968054 PMC7511409

[115] Li Y Lin S An N. Hsa_circ_0009910: Oncogenic Circular RNA Targets microRNA-145 in Ovarian Cancer Cells. Cell Cycle (2020) 19:1857–68. doi: 10.1080/15384101.2020.1731650 PMC7469526 32588730

[116] Wang H Xiao Y Wu L Ma D. Comprehensive Circular RNA Profiling Reveals the Regulatory Role of the circRNA-000911/miR-449a Pathway in Breast Carcinogenesis. Int J Oncol (2018) 52:743–54. doi: 10.3892/ijo.2018.4265 PMC5807038 29431182

[117] Chen T Yu Q Xin L Guo L. Circular RNA Circc3p1 Restrains Kidney Cancer Cell Activity by Regulating miR-21/PTEN Axis and Inactivating PI3K/AKT and NF-κB Pathways. J Cell Physiol (2020) 235:4001–10. doi: 10.1002/jcp.29296 31643094

[118] Huang H Wei L Qin T Yang N Li Z Xu Z. Circular RNA ciRS-7 Triggers the Migration and Invasion of Esophageal Squamous Cell Carcinoma *via* miR-7/KLF4 and NF-κB Signals. Cancer Biol Ther (2019) 20:73–80. doi: 10.1080/15384047.2018.1507254 30207835 PMC6343722

